# The effect of childhood and current economic status on depressive symptoms in South Korean individuals: a longitudinal study

**DOI:** 10.1186/s12939-016-0402-0

**Published:** 2016-07-18

**Authors:** Woorim Kim, Tae Hyun Kim, Tae-Hoon Lee, Yeong Jun Ju, Eun-Cheol Park

**Affiliations:** Department of Public Health, Graduate School, Yonsei University, Seoul, Republic of Korea; Institute of Health Services Research, Yonsei University, Seoul, Republic of Korea; Graduate School of Public Heath, Yonsei University, Seoul, Republic of Korea; Department of Preventive Medicine, Yonsei University College of Medicine, Seoul, Republic of Korea; Department of Preventive Medicine & Institute of Health Services Research, Yonsei University College of Medicine, 50 Yonsei-ro, Seodaemun-gu, Seoul, 120-752 Republic of Korea

**Keywords:** Income inequality, Childhood income, Current income, Depressive symptoms, Mental health, Intergenerational material transfer, Health inequality

## Abstract

**Background:**

Mental health inequality along the economic strata is prominent in South Korea, particularly as intergenerational material transfer is becoming increasingly important in gaining economic status. Therefore, this study aimed to investigate the relationship between current and childhood economic status and depressive symptoms in adults aged 20 or above.

**Methods:**

This study used data from the Korean Welfare Panel Study (KOWEPS), 2010 to 2013. A total of 9,645 individuals aged 20 years or above without depressive symptoms in 2010 were analyzed. The effect of childhood and current economic status, categorized into low, middle, and high groups, on depressive symptoms was investigated using hierarchical logistic regression models. Depressive symptoms were measured using the Center for Epidemiological Studies Depression (CES-D 11) scale. Subgroup analysis was performed based on education level.

**Results:**

Compared to the middle current-middle childhood economic status group, the low-low group (OR: 1.88, CI: 1.61-2.20), low-middle group (OR: 1.68, CI: 1.43-1.98), and low-high group (OR: 1.64, CI: 1.34-2.01) were more likely to have depressive symptoms. The high-low group (OR: 0.68, CI: 0.55-0.84), high-middle group (OR: 0.67, CI: 0.56-0.81), and high-high group (OR: 0.45, CI: 0.27-0.75) were less likely to have depressive symptoms. This trend was generally maintained with regard to education level, but the effects were not statistically significant in the high current economic status groups among participants with a university degree or above.

**Conclusion:**

Low current economic status was associated with a higher likelihood of depressive symptoms. In particular, the low current-low childhood economic status group showed the highest likelihood of depressive symptoms, suggesting the adverse mental health effects of prolonged poverty. Therefore, the findings reveal that mental health inequalities are present along the economic strata and require proper addressing of the mental health of lower income individuals.

## Background

Depression is an important public health problem predicted to become the most common cause of disability internationally by 2020 [1]. It is a noticeable burden to disability-adjusted life years (DALYs) in South Korea and has been ranked as one of its top leading causes [2]. Depression is particularly significant as the prevalence of major depressive disorder has persistently increased from 4.2 % in 2001 to 6.7 % in 2011 in South Korea [3]. The fact that the prevalence of adolescent depression has been reported to be as high as 20 % further adds importance to this subject [4]. Moreover, depression has also been associated with suicide attempts as around 60% of suicide deaths have been associated with mood and major depressive disorders [5]. South Korea ranks first among the Organization for Economic Cooperation and Development (OECD) countries in suicide rate and suicide is the fourth leading cause of mortality [6]. Naturally, depression is one of the main societal concerns in South Korea and it is important to identify and address the associated factors.

Socioeconomic status (SES) has been widely related to an increased likelihood of depressive symptoms. Among the factors that constitute SES, household income has been specifically associated with depressive symptoms in both adults and adolescents [7, 8]. Regarding the economic status of an individual, studies have shown that a family’s transfer of money often continues throughout the life course of a child and leads to the reproduction of income inequality because there are positive associations between parents’ income and the prospect of intergenerational material transfer [9]. Such findings are significant because income inequality has risen noticeably since the 1997 economic crisis in South Korea, with income related health inequality escalating as low SES groups show a disproportionate level of ill health [10, 11]. Hence, it is essential to understand how current and childhood economic status affect the depression levels of individuals because financial deprivation has been known to be associated with higher risks of depression throughout the life course of an individual.

Apart from household income, education level has also been reported to have a strong effect on the production of health inequality in South Korea. The South Korean society shows interesting contrasts in social status, with education level serving as an important factor that defines social class [12]. Education level has been closely associated with prestigious occupational positions, reflecting an important aspect of SES [13]. As family poverty has also been related to lower academic achievement in childhood, economic status and education level may concurrently affect the mental health of individuals in the South Korean society [14]. Therefore, the aim of this study was to investigate the association between current and childhood economic status, measured using household income, and depressive symptoms in individuals aged 20 or above and to further analyze how education level interplays in the objected relationship.

## Methods

### Study population

Korean Welfare Panel Study (KOWEPS) data for years 2010 to 2013 were used in this study. The KOWEPS is conducted by the Korean Institute for Health and Social Affairs, in conjunction with the Social Welfare Research Institute of Seoul National University, on a nationally representative sample of South Korean households [15]. The KOWEPS selects households based on a stratified, multistage, probability design and because it is nationally representative data, households are selected from the 16 provincial districts in proportion to the population size of each district. Households from urban and rural areas are included and all individuals aged 15 or above from the selected households are interviewed. Interviews are conducted annually during January to February using a computer assisted personal interviewing (CAPI) technique. Topics included in the KOWEPS are social service needs, healthcare utilization patterns, economic and demographic backgrounds, sources of income, subjective health status, and behavioral health status.

The 2010 data originally included 15,625 individuals. After excluding individuals aged 19 or below, 12,212 individuals were identified. From this population, 518 individuals with missing values on depressive symptoms and 236 individuals with missing values on other independent variables were excluded. Of the 11,458 remaining individuals, only study participants without depressive symptoms based on the Center for Epidemiological Studies Depression scale (CES-D 11) cutoff score in 2010 were included and followed for analysis. This led to the inclusion of 9,645 individuals in 2011 to form the baseline population. Afterwards, 9,402 individuals in 2012 and 9,127 individuals in 2013 were followed up in the analysis.

### Measures

#### Depressive symptoms

The outcome variable of this study was depressive symptoms, measured using the Korean version of the Center for Epidemiological Studies Depression scale (CES-D 11). The Korean version of the CES-D 11 has been utilized in many previous studies investigating depressive symptoms in the South Korean population [16]. The CES-D 11 is composed of 11 items scored based on the past week using a four point Likert scale. The Likert scale 0: ≤ 1 day per week; 1: 2–3 days per week; 2: 4–5 days per week; 3: ≥ 6 days per week is used for the following nine items, which are [a] “No appetite;” [b] “I felt quite depressed;” [c] “I felt difficulty in everything I did;” [d] “I could not sleep well;” [e] “I felt lonely;” [f] “I felt that people were treating me coldly;” [g] “My heart felt sad;” [h] “I felt that people disliked me;” and [i] “I was unable to have the courage to carry out something.” Two items [j] I felt that I was doing generally well and [k] I went on without much complaint are scored inversely (0: ≥ 6 days per week; 1: 4–5 days per week 2: 2–3 days per week 3: ≤ 1 day per week). Higher scores depict poorer mental health status and the resulting scores are multiplied by (20/ 11) to be comparable to the international standard CES-D 20 score [15]. The CES-D 11 is known to parallel the CES-D 20 standard version, and a score of 16 or above indicates probable depression [17, 18]. The KOWEPS also recommends a cutoff score of 16 out of 60 after transformation for the Korean version of the CES-D 11 [15].

#### Childhood and current economic status

The independent variable of this study, which is the combination of current and childhood economic status, was measured using separate questions. Childhood economic status was based on the question: “What was your family’s economic status during childhood?” The available responses were on a 5-point scale that ranged from very poor, poor, mediocre, affluent, to very affluent. Responses were then categorized as low, medium, or high. Current economic status was measured using household income, which encompasses ordinary (wage and salary income, gross self-employment income, realized property income, occupational pensions, social insurance income, and social aid income) and non-ordinary (inheritance and income from asset disposals) household income. Equalized household income is calculated by dividing the household income by the square root of the number of family members, which is the standard method proposed by the OECD. Using equalized household income gives the advantage of allowing comparability between households of different sizes [19]. Current economic status was then categorized as low, medium, or high based on reported yearly average wages in South Korea. Afterwards, childhood economic status and current economic status were combined into the low-low, low-middle, low-high, middle-low, middle-middle, middle-high, high-low, high-middle, and high-high categories.

#### Covariates

Demographic, socioeconomic, and health-related covariates were included in this study. The included covariates were age (20–29 years, 30–39 years, 40–49 years, 50–59 years, 60–69 years, and 70 years or above), gender (male and female), education level (middle school, high school, and university or above), marital status (single and married), employment status (economically inactive and economically active), family satisfaction level (low, medium, and high), perceived health status (low, medium, and high), and chronic disease status (none and one or above).

### Statistical analysis

Chi square tests were performed to examine the study participants’ general characteristics and compare differences between groups. Hierarchical logistic regression model using the GLIMMIX procedure was utilized in examining the association between current and childhood economic status and depressive symptoms. Hierarchical logistic regression models were used because the data used in this study was longitudinal and hierarchically organized. The KOWEPS data included households selected using a stratified multistage probability design and contained multiple individuals from the same households, inferring that annual repeated measurements of the same individuals were present. Subgroup analysis was conducted on education level through an interaction test completed between current and childhood economic status and education level. The calculated *P*-values in this study were two-sided and considered significant at less than 0.05. All analysis was conducted using the SAS software, version 9.4 (SAS Institute, Cary, NC, USA).

## Results

Table 1 presents the general characteristics of the study participants at the 2011 baseline. The results show 1,552 (16.1 %) individuals with depressive symptoms. Higher proportions of individuals with depressive symptoms can be seen in the low current economic status group categories, followed by the middle and high current economic status group categories. Specifically, the low current-low childhood economic status group had the highest percentage of individuals with depressive symptoms (33.1 %) whereas the high current-high childhood economic status group had the lowest percentage of individuals with depressive symptoms (4.0 %)

**Table 1:** General characteristics at first follow-up (2011) of study participants without depressive symptoms in 2010

				N (%)
	N	Depressive symptoms		*p*-value
		No	Yes	
Current-childhood economic status				
Low-low	1789	1197 (66.9)	592 (33.1)	<.0001
Low-middle	1117	826 (74.0)	291 (26.1)	
Low-high	486	338 (69.6)	148 (30.5)	
Middle-low	1316	1140 (86.6)	176 (13.4)	
Middle-middle	1494	1348 (90.2)	146 (9.8)	
Middle-high	356	322 (90.5)	34 (9.6)	
High-low	1035	969 (93.6)	66 (6.4)	
High-middle	1678	1602 (95.5)	76 (4.5)	
High-high	374	359 (93.9)	15 (4.0)	
Gender				
Male	4196	3716 (88.6)	480 (11.4)	<.0001
Female	5449	4377 (80.3)	1072 (19.7)	
Education level				
Middle school	4433	3310 (74.7)	1123 (25.3)	<.0001
High school	2802	2515 (89.8)	287 (10.2)	
University or above	2410	2268 (94.1)	142 (5.9)	
Marital status				
Single	151	140 (92.7)	11 (7.3)	0.0030
Married	9494	7953 (83.8)	1541 (16.2)	
Employment status				
Economically non-active	4467	3416 (76.5)	1051 (23.5)	<.0001
Active	5178	4677 (90.3)	501 (9.7)	
Family satisfaction level				
Low	482	228 (47.3)	254 (52.7)	<.0001
Medium	1537	1093 (71.1)	444 (28.9)	
High	7626	6772 (88.8)	854 (11.2)	
Perceived health status				
Low	2640	1699 (64.4)	941 (35.6)	<.0001
Medium	2375	2034 (85.6)	341 (14.4)	
High	4630	4360 (94.2)	270 (5.8)	
Chronic disease status				
None	4516	4159 (92.1)	357 (7.9)	<.0001
1 or above	5129	3934 (76.7)	1195 (23.3)	
Total^a^	9645	8093 (83.9)	1552 (16.1)	

^a^Age (mean, standard deviation) = 55.5, 16.85

The results of the hierarchical logistic regression models analyzing the effect of current and childhood economic status on depressive symptoms are shown in Table 2. When setting the middle current-middle childhood economic status group as a reference, the low current-low childhood, low current–middle childhood, and low current-high childhood groups were more likely to have depressive symptoms. Contrastingly, the high current-low childhood, high current-middle childhood, and high current-high childhood groups were less likely to exhibit depressive symptoms.

**Table 2:** Factors associated with depressive symptoms in study participants

	OR^a^	95 % CI
Current-childhood economic status		
Low-low	1.88	(1.61 – 2.20)
Low-middle	1.68	(1.43 – 1.98)
Low-high	1.64	(1.34 – 2.01)
Middle-low	1.16	(0.98 – 1.37)
Middle-middle	Ref	–
Middle-high	0.98	(0.76 – 1.26)
High-low	0.68	(0.55 – 0.84)
High-middle	0.67	(0.56 – 0.81)
High-high	0.45	(0.27 – 0.75)
Age	1.01	(1.00 – 1.01)
Gender		
Male	Ref	–
Female	1.56	(1.42 – 1.72)
Education level		
Middle school	Ref	–
High school	0.94	(0.82 – 1.08)
University or above	0.87	(0.73 –1.03)
Marital status		
Single	Ref	–
Married	1.04	(0.68 – 1.59)
Employment status		
Economically non-active	Ref	–
Active	0.75	(0.68 – 0.83)
Family satisfaction level		
Low	Ref	–
Medium	0.46	(0.40 – 0.54)
High	0.20	(0.18 – 0.24)
Perceived health status		
Low	Ref	–
Medium	0.37	(0.33 – 0.41)
High	0.22	(0.20 – 0.25)
Chronic disease status		
None	Ref	–
1 or above	1.02	(0.90 – 1.14)
Year		
2011	Ref	–
2012	0.99	(0.79 – 1.36)
2013	0.97	(0.71 – 1.43)

^a^Adjusted for sex, age, marital status, education level, employment status, family satisfaction level, perceived health status, chronic disease status, and year

Lastly, Table 3 presents the results of the hierarchical logistic regression models analyzing the effect of current and childhood economic status on depressive symptoms by education level. The trends shown in Table 2 were generally maintained and compared to the middle current-middle childhood reference group, the low current-low childhood, low current–middle childhood, and low current-high childhood groups had increased likelihoods of depressive symptoms whereas the high current-low childhood, high current-middle childhood, and high current-high childhood groups had decreased likelihoods of depressive symptoms. However, in the university or above group, only the low current-low childhood and low current-high childhood groups showed a statistically significant higher likelihood of depressive symptoms.

**Table 3:** Results analyzing the effect of current and childhood economic status by education level

		OR^a^	95 % CI
Education level			
Middle school	Low-low	1.84	(1.48 – 2.28)
	Low-middle	1.73	(1.38 – 2.18)
	Low-high	1.49	(1.13 – 1.96)
	Middle-low	1.21	(0.96 – 1.54)
	Middle-middle	Ref	
	Middle-high	0.92	(0.63 – 1.36)
	High-low	0.64	(0.46 – 0.88)
	High-middle	0.66	(0.46 – 0.95)
	High-high	0.40	(0.14 – 1.14)
High school	Low-low	2.05	(1.48 – 2.85)
	Low-middle	1.67	(1.24 – 2.25)
	Low-high	1.95	(1.29 – 2.95)
	Middle-low	1.07	(0.79 – 1.46)
	Middle-middle	Ref	
	Middle-high	1.03	(0.66 – 1.60)
	High-low	0.68	(0.47 – 0.97)
	High-middle	0.52	(0.38 – 0.72)
	High-high	0.30	(0.11 – 0.83)
University or above	Low-low	2.23	(1.33 – 3.76)
	Low-middle	1.23	(0.77 – 1.97)
	Low-high	2.52	(1.43 – 4.42)
	Middle-low	0.93	(0.57 – 1.51)
	Middle-middle	Ref	
	Middle-high	1.25	(0.69 – 2.24)
	High-low	0.81	(0.51 –1.29)
	High-middle	0.87	(0.63 –1.22)
	High-high	0.98	(0.63 – 1.68)

^a^Adjusted for sex, age, marital status, employment status, family satisfaction level, perceived health status, chronic disease status, and year

## Discussion

The results of this study show that individuals with low current economic status had increased likelihoods of depressive symptoms compared to individuals in the middle current-middle childhood economic status group. On the other hand, individuals with high current economic status had decreased likelihoods of depressive symptoms than individuals in the middle current-middle childhood economic status group. This suggests that current economic status measured using household income can have a marked effect on depressive symptoms. The findings are also particularly significant because the low current-low childhood economic status group showed the highest likelihood of depressive symptoms, inferring the adverse mental health effects of prolonged poverty. South Korea achieved rapid industrialization and economic development since the 1960s, with its per capita gross national income escalating from US $100 in 1965 to greater than US $20,000 in 2010 [20]. However, South Korea was not immune to the economic crisis that impacted many Asian countries in 1997 and economic inequality has persistently grown due to the adoption of flexible labor markets and a lack of a provision of a stable social safety net [21]. In fact, the disposable income Gini coefficient of South Korea has continuously increased and the proportion of the middle class has declined from 70.7 % in 1994 to 56.0 % in 2005 [22]. Studies have shown that socioeconomically disadvantaged groups have disproportionately higher levels of ill health and increased risks of depression. There are concerns that the increasing economic inequalities in South Korea may contribute to higher levels of depression among low income groups [11, 23]. Low material standards have also been associated with an increased risk of depression [24]. A study by Hong et al. in 2011 demonstrated a notable pro-rich inequality regarding depression including suicide ideation and suicide attempts, which doubled over a 10 year period from 1997 to 2007 [25]. It has been suggested that this may be due to low social status being associated with increased stress; individuals often having less control of their lives and at a higher risk of experiencing uncontrollable threats to their social esteem as they perceive being looked down upon and devalued [26]. Therefore, the mental health effects of low economic status can be pronounced because income serves as a measure of social class and consumption patterns clarify the distinction between the upper and lower classes [22].

When reporting on current and past SES it is also important to consider the significance of intergenerational monetary transfer. The findings of this study reveal that differences in depressive symptoms are highly affected by current economic status. However, the extent of change also seems to be affected by childhood economic status as the low current-low childhood group shows higher a higher likelihood of depressive symptoms than the low current-high childhood group. Similarly, the high current-high childhood group has a lower odds ratio than the high current-low childhood group. This implies that childhood economic status may have lasting effects on depressive symptoms, although current economic status is most predominantly related. In fact, studies have revealed that a downward transfer of financial resources from older to younger generations are common and that bequests can perpetuate income inequality [27, 28]. Parents of high SES also often tend to heavily invest in their offspring in the belief that financial investments will help their offspring achieve success and attain higher SES as adults [29]. Since inheritance is known to contribute noticeably to attaining wealth in South Korea because of its ageing population and lowered economic growth, it can be inferred that offspring of lower income groups face increased difficulties in reaching higher economic status [30]. Therefore, it is critical to address the negative mental health effects of income inequality because chronic strain resulting from economic hardship has been associated with depression [14].

Although income is a significant marker of SES other important socioeconomic factors to consider include education level and occupation [31]. Specifically, education level may impact the relationship between current and childhood economic status and depressive symptoms because education has been strongly associated with occupation and financial status in South Korea [13]. While education level alone may be considered by some to be a reflection of high social status and prestige, it is also an undeniably important factor when it comes to attaining reasonable future employment and security, which will naturally impact general wellbeing. This study also presents that the effect of economic status on depressive symptoms is more pronounced in the middle and high school graduate groups than individuals with a university degree or above. In the university graduate group, high current income groups do not show a decreased likelihood of depressive symptoms compared to the middle current income groups. Previous studies conducted in South Korea have also found similar results, in which higher education level was related to lower levels of depressive symptoms [1]. This implies that education level can help moderate the negative mental health effects of lower income. Yet parental economic status has been associated with increased parental support through shadow education and better academic achievement in South Korea. Parents of low income families often experience burdens in academically supporting their children and many comparatively disadvantaged offspring find themselves lagging behind. Therefore, because economic and education status can both affect depressive symptoms, it is important to increase awareness about the relationship between socioeconomic inequality and mental health [32–34].

In conclusion, this study shows that mental health inequalities are present along the socioeconomic stratum in South Korea. The South Korean government has implemented the New Health Plan 2010 in order to improve national health equity by addressing the worsening gaps in education, employment status, and income [25]. However, this plan only encompasses health equity in two major aspects: mortality and health behavior [21]. Mental health is not included despite the fact that depression is the second leading cause of morbidity in industrialized countries and more prevalent among socioeconomically disadvantaged groups [35, 36]. Therefore, a modified health policy that encompasses the most vulnerable diseases is required to reduce health inequality. Furthermore, because East Asians generally express similar patterns in the perception and expression of mental disorders, the revealed factors leading to mental health inequality can be more generally applied [37, 38].

This study has some limitations. First, there may have been recall bias because childhood economic status was measured using self-reports due to data limitations. Additionally, because childhood economic status was measured using a single question, responses may have been subjective. Future studies using objective criteria for childhood economic status are needed to provide further insights. Second, although this study was longitudinal in design, the possibility of reverse causality cannot be completely removed as there may have been bidirectional relationships between current and childhood economic status, education level, and depressive symptoms. Lastly, this study categorized education level into middle school, high school, and university or above without further distinguishing between undergraduate and postgraduate degrees. This may have underestimated the presented results. Despite these limitations, this study is unique in that it incorporates the effect of both current and childhood economic status on depressive symptoms while accounting for education level in the aim of revealing mental health inequalities present along the socioeconomic strata.

## Conclusion

The results of this study show an association between current and childhood economic status and increased likelihoods of depressive symptoms, demonstrating that mental health inequalities are present along the socioeconomic strata. Education level also has an influence on the stated relationship as the effect of economic status on depressive symptoms was comparatively pronounced in middle and high school graduates. Therefore, sufficient financial resources for mental health services should be provided to economically disadvantaged groups to ensure access opportunities.

## Abbreviations

ANOVA, analysis of variance; CES-D, center for epidemiologic studies depression; DALY, disability adjusted life year; KOWEPS, Korean welfare panel study; OECD, organization for economic development and cooperation
